# Association Between Cardiovascular Disease and Chronic Kidney Disease Prevalence and Characteristics in Saudi Arabia

**DOI:** 10.7759/cureus.50205

**Published:** 2023-12-08

**Authors:** Ahmed A Elheet, Mohammed A Alosaimi, Waad A Alalawi, Awadh A Alasmari, Aeshah Alharbi, Lama Alhumaidan, Reuof S Alosaimi, Riyadh A Alharthi, Hameedullah M Kazim

**Affiliations:** 1 Cardiology Department, Alhada Armed Forces Hospital, Taif, SAU; 2 College of Medicine, Taif University, Taif, SAU; 3 College of Medicine, Unaizah College of Medicine and Medical Sciences, Qassim University, Unaizah, SAU; 4 College of Medicine, Ibn Sina National College for Medical Studies, Jeddah, SAU; 5 Cardiac Surgery, King Abdulaziz Specialist Hospital, Taif, SAU

**Keywords:** cardiovascular disease, association, estimated glomerular filtration rate, ejection fraction, chronic kidney disease

## Abstract

Introduction

Cardiovascular disease (CVD), including coronary artery disease (CAD), is a leading global cause of death. Chronic kidney disease (CKD) is a significant risk factor, particularly in data-scarce Saudi Arabia, due to shared risk factors. A study aims to assess the CVD-CKD relationship, identifying clinical characteristics and risk factors to improve prevention and care in this context, filling a knowledge gap in Saudi Arabia's healthcare map.

Methodology

It is a single-center retrospective study aimed at evaluating the relationship between cardiovascular disease and chronic kidney disease, conducted between January 2023 and October 2023. Data was sourced from patient files using a data sheet based on a previous study. The data was cleaned in MS Excel (Redmond, USA) and analyzed in IBM Corp. Released 2022. IBM SPSS Statistics for Windows, Version 29.0. Armonk, NY: IBM Corp.

Results

Our study contains predominantly males (61%), aged 61-80 (54.1%), with a normal body mass index (BMI) (<25) (61.5%) and a high prevalence of smoking (72.3%). Diabetes, hypertension, and smoking were prevalent risk factors. The relationship between CAD severity, renal dysfunction, and ejection fraction (EF) was explored, emphasizing the association between declining renal function and more advanced CAD stages, as well as the decline in estimated glomerular filtration rate (eGFR) with decreasing EF. Age, smoking, CAD, and decreasing EF were linked to renal dysfunction, while smoking, stroke history, peripheral vascular disease (PVD), BMI, and decreasing EF were associated with CAD stage severity.

Conclusion

Our study explored that as CAD severity increases, renal function decreases, showing both CVD and CKD connected with each other, and a similar correlation occurs between decreasing EF and decreasing eGFR, revealing significant associations with various risk factors. Further research is warranted to explore potential interventions aimed at mitigating the synergistic impact of CVD and CKD on patient morbidity and mortality.

## Introduction

Coronary artery disease (CAD) is the most prevalent kind of cardiovascular disease (CVD), which is one of the leading causes of death worldwide. Worldwide, CVD caused 19 million deaths in 2020 [1]. Prior research has demonstrated a connection between CVD risk factors and chronic kidney disease (CKD). Furthermore, the risk of morbidity and mortality from CVD is significantly higher in patients with CKD [2]. There is not enough information available about the prevalence of CKD and CVD in Saudi Arabia. According to estimates, the prevalence of CVD in Saudi Arabia is 5.5% [3]. This is because risk factors for both CVD and CKD, such as smoking, diabetes, hypertension, and hyperlipidemia, are identical. Furthermore, atherosclerosis, which is recognized as one of the most frequent causes of CVD, also affects the renal vasculature [4-6]. It is typical to have both CVD and CKD concurrently for this reason [7]. More than 20,000 patients in Saudi Arabia are on dialysis at the moment, and 9,810 kidney transplant recipients are receiving follow-up care [8]. Individuals with end-stage renal disease have a risk of cardiovascular disease (CVD) that is 10-20 times higher than the general population, and CVD is the cause of 50% of deaths in this population [9,10].

In a 2006 study comparing people with and without chronic kidney disease (CKD) to determine the prevalence of CVD and risk factors for it, the researchers discovered that people with CKD were more likely to have hypertension and to have poorly controlled blood pressure. Furthermore, they discovered that among them, elevated low-density lipoprotein cholesterol levels were more common. Diabetes and other risk factors were shown [7]. Additionally, a different cohort study that was carried out in Taiwan to evaluate this association shows that patients with CVD have a 4.1-fold increased incidence of CKD and are also more likely to have diabetes, hypertension, and CAD. Additionally, for the course of treatment, comparing participants with and without CKD, those with CKD had more difficulty controlling their blood pressure. Despite having a higher median number of antihypertensive medications than participants without CKD, hypertensive participants with CKD had poorly controlled blood pressure, with nearly twice as many receiving treatment for it. This may indicate that the severity of the CVD risk factors may be elevated by CKD [11]. Considering that not enough is known about this relationship, particularly in Saudi Arabia.

Our study's objective is to evaluate the connection between CKD and CVD. Clinical features and CKD prevalence among CVD patients will be part of our evaluation. Additionally, as far as we are aware, no prior research has evaluated this relationship in Saudi Arabia. Our research may be useful in identifying these clinical traits and in developing more protective measures to identify, track, and prevent CVD in CKD and vice versa. Finding risk factors for obesity, diabetes, atherosclerosis, and hypertension, among others, may help achieve this. Finding these clinical traits and associated risk factors may enhance the prognosis for CVD and CKD while also promoting more investigation into the etiology of these two conditions that frequently coexist. This study aims to evaluate the relationship between cardiovascular disease (prevalence and characteristics) and chronic kidney disease.

## Materials and methods

Study design

This single-center retrospective study was conducted in Al-Hada Armed Forces Hospital, Taif, Saudi Arabia, between January and October 2023, with the goal of evaluating the relationship between cardiovascular disease and chronic kidney disease. 

Study population and sampling methodology

One thousand eleven hundred and eighteen patients made up the study's sample, and the information was taken from patient records at the Alhada Armed Forces Hospital in Taif City, Saudi Arabia. The study included all patients with chronic kidney disease (CKD) who were between the ages of 18 and 75, had been admitted with unstable angina or myocardial infarction, or had chest pain and a positive treadmill test or imaging (ECHO or DSE), or were candidates for a renal transplant but had multiple risk factors. The study ran from January 2015 to December 2022. Every patient who is younger than 18 or older than 75, has a severe allergy to iodinated contrast, is actively bleeding, has a known bleeding diathesis, has a known coagulation disorder, has a platelet count of less than 100,000/mm3 or an international normalized ratio greater than 2.5 upon admission, or has undergone cardiopulmonary resuscitation for more than 10 minutes, or breastfeeding or pregnancy, or active cancer, or has a life expectancy less than six months. Patients who were diagnosed outside of the study period or who lacked clinical data in their medical records were excluded from the study due to their inability to comply with study assessments. Data were collected through a previously validated study by Milane A. in 2015 [12].

Statistical analysis

Data entry and analysis were conducted using IBM Corp. Released 2022. IBM SPSS Statistics for Windows, Version 29.0. Armonk, NY: IBM Corp. In addition to the frequency and percentages of each independent variable, descriptive statistics like the mean score and standard deviation were also used. The responses were tallied according to frequency and percentage, which were subsequently translated into percentage mean scores and, as previously indicated, converted into qualitative data. The right analytical statistics were used. A p-value of less than 0.05 is deemed significant.

Ethical considerations

The Institutional Review Board (IRB) of the Alhada Armed Forces Hospital in Taif granted ethical approval (REC.2022-689). Because the study was retrospective in nature, written informed consent was not required, and it was conducted in compliance with the Declaration of Helsinki.

## Results

One thousand eleven hundred and eighteen patients participated in our study. 61.5% of them had a normal BMI (<25), the majority were male (61%), and the majority were between the ages of 61 and 80 (54.1%), according to Table 1. 72.3 percent of patients had a history of smoking. 5.5% of cases had mortality within the previous 30 days, and 8.1% of cases had reported hospital deaths.

**Table 1:** Sociodemographic characteristics of patients (n=1118) BMI: Body mass index

Variable	Categories	Frequency (N)	Percent (%)
Age (Years)	31-40	45	4.0
	41-50	78	7.0
	51-60	101	9.0
	61-70	241	21.6
	71-80	363	32.5
	> 80	290	25.9
Gender	Male	682	61.0
	Female	436	39.0
BMI	< 25 (Normal)	688	61.5
	25 - 30 (Overweight)	307	27.5
	31-35 (Class 1 Obese)	117	10.5
	> 35 (Class 2 Obese)	6	0.5
Smoking History	Non- Smokers	234	20.9
	Smoker	808	72.3
	Ex-Smokers	76	6.8
Hospital Deaths	No	1027	91.9
	Yes	91	8.1
Last 30 Days Mortality	No	1056	94.5
	Yes	62	5.5

**Figure 1:**
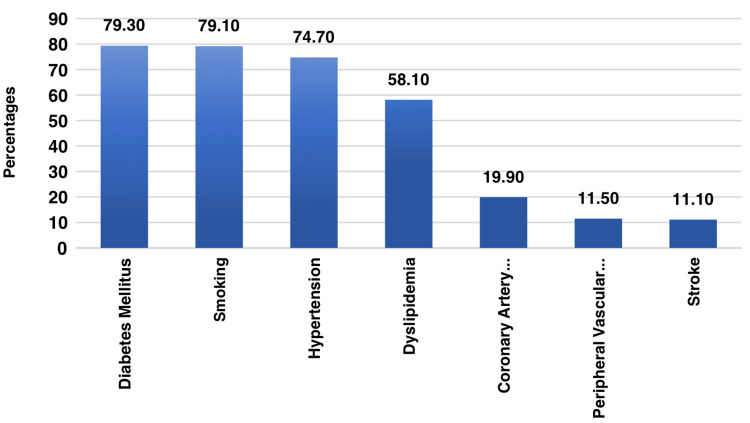
Different risk factors/Comorbidities associated with CVD/CKD (n=1118) CVD: Cardiovascular disease, CKD: Chronic kidney disease

The prevalence of different risk factors and comorbidities in people with CVD/CKD is depicted in Figure 1. Smoking and diabetes mellitus, which affect 79.1% and 79.3% of the population, respectively, are two extremely common risk factors. While coronary artery disease and stroke are less common (19.9% and 11.1%, respectively), hypertension is also common (74.7%).

A thorough summary of the characteristics of chronic kidney disease (CKD) and cardiovascular disease (CVD) in people is provided in Table 2. Notably, among the patients, non-ST elevation MI accounted for 33.5%, unstable angina for 7.2%, ST elevation MI for 29.9%, and other cardiac conditions for 29.4%. Furthermore, 19.9% reported a history of coronary artery disease (CAD), with a range of severity from very severe (21.6%) to normal (13.9%). 6.4% of patients had a bypass graft, and 11.4% had percutaneous interventions, indicating prior revascularization procedures. ACEI and ARBs (81.6%), mineralocorticoid receptor agonists (35.5%), and angiotensin receptor neprilysin inhibitors (47.9%) were among the medications. Of the patients, 42.5% had abnormal BNP levels, and 65.6% had abnormal troponin levels. Different eGFR levels were shown by CKD parameters; 9.1% of patients were on dialysis, and 9.1% were transplant candidates. Finally, 45.6% were using SGLT2I medication.

**Table 2:** CVD and CKD features of patients (n=1118) ACEI: ACE inhibitors, ARBs: Angiotensin receptors blockers, SGLT2I: Sodium glucose type 2 transporter inhibitors, eGFR: Estimated glomerular filtration rate, BNP: Brain natriuretic peptide

Variable	Categories	Frequency (N)	Percent (%)
Suffering from CVD	ST Elevation MI	334	29.9
	Non- ST Elevation MI	374	33.5
	Un Stable Angina	81	7.2
	Other (Chest Pain etc.)	329	29.4
Hx of CAD	No	895	80.1
	Yes	223	19.9
Left main artery occlusion	No	1018	91.1
	Yes	100	8.9
CAD severity based on severity scales (SS)	Normal (0)	155	13.9
	Mild Severity (1-22)	272	24.3
	Moderate Severity (22-32)	347	31.0
	Very Severe (> 32)	242	21.6
	Thrombosis	102	9.1
Previous revascularization	No	901	80.6
	Coronary Artery Bypass Graft	72	6.4
	Percutaneous Interventions	127	11.4
	Both	18	1.6
Medication for CVD	ACEI & ARBS	912	81.6
	Mineralocorticoid Receptor Antagonist (Spironolactone)	397	35.5
	Angiotensin Receptor Neprilysin Inhibitor (Entresto)	536	47.9
Troponin	Normal	385	34.4
	High	733	65.6
BNP	Normal	643	57.5
	High	475	42.5
CKD parameters			
eGFR of patients (ml/min)	90-120	434	38.8
	60-89	402	36.0
	15-29	180	16.1
	< 15	102	9.1
Dialysis history	No	1016	90.9
	Yes	102	9.1
Transplant candidate	No	1016	90.9
	Yes	102	9.1
SGLT2I (Empagliflozin)	No	608	54.4
	Yes	510	45.6

The adjusted odds ratios (OR) for the outcome variable of renal dysfunction (RDL, defined as eGFR < 90 ml/min) are displayed in Table 3. Among the noteworthy results are the significant positive association of age (OR = 1.027, 95% CI: 1.01-1.03) and the significant reduction in odds associated with quitting smoking (OR = 0.304, 95% CI: 0.16-0.56). The same is true for BMI (overweight and obese classes) and diabetes (DM) (OR = 0.674, 95% CI: 0.47-0.96). Interestingly, CAD and renal dysfunctions are strongly positively correlated (OR = 3.133, 95% CI: 2.04-4.80), and lower EF is associated with increased odds of renal dysfunction (OR = 1.346, 95% CI: 1.18-1.53) of RDL.

**Table 3:** Adjusted odds ratios predicting RDL (eGFR < 90 ml/min) as an outcome variable eGFR: Estimated glomerular filtration rate, RDL: Renal dysfunction levels, BMI: Body mass index, DM: Diabetes mellitus, HTN: Hypertension

Variable	B	Sig.	Exp(B)	CI 95%
Age (Years)	.027	<0.001	1.027	1.01-1.03
Gender (Female)	-.306	.056	.737	0.53-1.00
Smoking (Non)	REF	<0.001	REF	
Smoking (current)	.044	.818	1.045	0.71-1.51
Smoking (Ex)	-1.192	<0.001	.304	0.16-0.56
HTN	.103	.527	1.109	0.80-1.52
DM	-.395	0.029	.674	0.47-0.96
Dyslipidemia	.705	<0.001	2.025	1.49-2.73
Stroke	.763	0.036	2.145	1.04-4.38
PVD	-.151	.669	.859	0.42-1.72
CAD	1.142	<0.001	3.133	2.04-4.80
BMI (Normal)	REF	<0.001	REF	
BMI (Overweight)	-1.687	<0.001	.185	0.13-0.26
BMI (Obese Class 1)	-1.139	<0.001	.320	0.17-0.157
BMI (Obese Class 2)	18.241	.999	83594578.943	.000
Increasing Severity of CAD on SS	.075	.244	1.078	0.95-1.22
Decrease EF	.297	<0.001	1.346	1.18-1.53
Constant	-1.909	<0.001	.148	

The adjusted odds ratios (OR) for the outcome variable of increasing coronary artery disease (CAD) severity are displayed in Table 4. Among the noteworthy results are the strong positive correlation between smoking (current) and CAD (OR = 2.583, 95% CI: 1.62-4.09) and the significant reduction in the odds associated with a history of stroke (OR = 0.055, 95% CI: 0.01-0.15). There is a strong correlation between peripheral vascular disease (PVD) and a higher risk of CAD (OR = 13.184, 95% CI: 4.20-41.3). Both rising BMI (OR = 1.545, 95% CI: 1.08-2.20) and falling ejection fraction (EF) (OR = 1.989, 95% CI: 1.62-2.47) are positively associated with increased odds of developing CAD.

**Table 4:** Adjusted odds ratios predicting CAD (increasing severity) as an outcome variable EF= Ejection fraction of the left ventricle, PVD: Peripheral vascular disease, DM: Diabetes mellitus, HTN: Hypertension

Variable	B	Sig.	Exp(B)	CI 95%
Age (Years)	.012	.083	1.012	0.99 - 1.02
Gender (Female)	-.062	.772	.940	0.61 - 1.43
Smoking (Non)	REF	<0.001	REF	-
Smoking (current)	.949	<0.001	2.583	1.62 - 4.09
Smoking (Ex)	20.273	.996	637600295.260	-
HTN	.043	.840	1.044	0.68 – 1.58
DM	-.041	.866	.960	0.59 – 1.55
Dyslipidemia	-.293	.149	.746	0.50 – 1.11
Stroke	-2.905	<0.001	.055	0.01 – 0.15
PVD	2.579	<0.001	13.184	4.20 – 41.3
Increasing BMI	.435	0.016	1.545	1.08 – 2.20
Decreasing EF	.688	<0.001	1.989	1.62 -2.47
Constant	-1.479	0.013	.228	

The distribution of patients by estimated glomerular filtration rate (eGFR) ranges in milliliters per minute (ml/min), and severity stages of coronary artery disease (CAD) is displayed in Table 5. Notably, the percentage of patients in severe-to-thrombosis CAD stages rises significantly as eGFR levels fall. For example, 72 patients (20.9%) with an eGFR < 15 ml/min were in the severe thrombosis CAD stage, demonstrating the association between the severity of CAD and renal dysfunction.

**Table 5:** Patients’ distribution by CAD stages and by levels of renal dysfunction defined by ranges of eGFR expressed in ml/min eGFR= Estimated glomerular filtration rate

Variable	CAD Severity Staging Based on Severity Score Scale					Sig. Value
	eGFR (ml/min)	N	Normal (n=155)	Mild to Moderate (n=619)	Severe to Thrombosis (n=344)	
Renal dysfunction levels expressed by ranges of eGFR (ml/min)	Normal (90-120)	434	77 (49.7)	259 (41.8)	98 (28.5)	<0.001
	Level 1 (60-89)	402	60 (38.7)	222 (35.9)	120 (34.9)	0.710
	Level 3 (15-29)	180	12 (7.7)	114 (18.4)	54 (15.7)	0.003
	Level 4 (< 15)	102	6 (3.9)	24 (3.9)	72 (20.9)	<0.001

The patients' distribution is displayed in Table 6 according to the ejection fraction (EF) of the heart's left ventricle (LV) and the degree of renal dysfunction indicated by estimated glomerular filtration rate (eGFR) ranges in milliliters per minute. Notably, there is a notable trend toward lower eGFR levels as heart EF declines. For example, 42 (13.0) and 30 (15.0%) of the patients with eGFR < 15 ml/min have an EF of 30%-40% and < 30%, respectively.

**Table 6:** Patients’ distribution by ejection fraction and by levels of renal dysfunction defined by ranges of eGFR expressed in ml/mil

Variable	Ejection Fraction of LV of the Heart						Sig. Value
	eGFR (ml/min)	N	> 50% (n=377)	40-50% (n=219)	30-40% (n=322)	< 30% (n=200)	
Renal dysfunction levels expressed by ranges of eGFR (ml/min)	Normal (90-120)	434	197 (52.3)	87 (39.7)	82 (25.2)	68 (34.0)	<0.001
	Level 1 (60-89)	402	132 (35.0)	66 (30.1)	132 (41.0)	72 (36.0)	0.075
	Level 3 (15-29)	180	42 (11.1)	42 (19.2)	66 (20.5)	30 (15.0)	0.004
	Level 4 (< 15)	102	6 (1.6)	24 (11.0)	42 (13.0)	30 (15.0)	<0.001

## Discussion

CVD, including CAD, is a top global cause of death, with CKD being a notable risk factor, particularly in Saudi Arabia, where data is scarce. Shared factors like diabetes and hypertension worsen this link, alongside atherosclerosis. Our study explored the intricate connection between CVD and CKD, focusing on risk factors, comorbidities, CAD severity, renal function, and left ventricle ejection fraction (EF) implications.

Our study's demographic analysis highlights that a significant proportion of participants were males, which shows that CKD and CVD may be gender-specific and that the prevalence of both is higher in males than females. This finding is consistent for CAD, which is more prevalent in males [13], but CKD is more prevalent in females [14]. Regarding age, old age, like 61-80 years, is more likely to be associated with CKD and CVD-related deaths [15,16]. Most of our participants are in the normal range of BMI (<25) (61.5%). The smoking history was remarkably prevalent, as most of the patients had a history of smoking. Furthermore, higher hospital deaths are associated with CKD and CVD.

Diabetes mellitus and smoking were prevalent among individuals with CVD/CKD, alongside hypertension, coronary artery disease, and stroke. This underscores the significant burden of traditional cardiovascular risk factors in our study population [17]. Importantly, there is a diverse range of CVD manifestations, including ST elevation MI and non-ST elevation MI. Surprisingly, a significant majority had no prior CAD history, but within the CAD group, severity varied considerably from normal to very severe. These findings emphasize the need for personalized management strategies due to the heterogeneous phenotypic presentation of CAD [18]. Notably, our findings align with contemporary cardiovascular treatment trends, as a significant portion of patients received ACEI and ARBs (81.6%), mineralocorticoid receptor antagonists (35.5%), and angiotensin receptor neprilysin inhibitors (47.9%). This reflects the adoption of evidence-based therapies [19, 20]. Abnormal troponin and BNP levels are consistent with literature emphasizing the need for vigilant monitoring and management of cardiac and renal function in this patient group to optimize outcomes. Thus, our findings reinforce the importance of comprehensive care for CVD/CKD patients in line with current guidelines.

A significant number of CKD patients were on dialysis, and an equal portion were transplant candidates. This corroborates existing literature emphasizing the adverse cardiovascular impact of CKD [21]. A comprehensive, dual-focus approach to cardiac and renal health is crucial, as recommended in prior research. Notable findings of our study revealed intriguing associations in predicting renal dysfunction, aligning with previous literature on cardiovascular and renal interplay. Age exhibited a positive correlation with renal dysfunction, consistent with aging's known impact on renal function. Surprisingly, being an ex-smoker was linked to lower odds of renal dysfunction, possibly reflecting smoking cessation benefits [22]. Diabetes showed a protective effect, which may be due to Reno protective drugs in DM, while CAD's strong association underscored their intricate relationship. Moreover, the link between decreasing EF and renal dysfunction reaffirms the intimate connection between cardiac and renal health, reinforcing existing knowledge in the field [23].

Several important insights into predictors of CAD severity corroborate existing research. Current smoking's strong association with higher CAD severity reaffirms its adverse impact on cardiovascular health. Surprisingly, a history of stroke showed a protective effect, possibly due to differing risk profiles or prophylactic antiplatelets and anticoagulant therapies. This is in contrast with the previous literature, which shows that stroke increases the risk of CAD [24]. PVD's robust link emphasizes the need for peripheral vascular assessment in CVD patients. Higher BMI's association underscores obesity's role in CAD progression. The correlation between reduced EF and CAD severity aligns with established knowledge, emphasizing the importance of cardiac function in CAD prognosis. Importantly, given the interconnectedness of cardiac and renal health in CVD/CKD patients, our findings align with previous research, highlighting the correlation between declining eGFR and more severe CAD stages. Similarly, lower EF causes a decrease in eGFR. These results emphasize the necessity of evaluating both heart and kidney function in this patient population, as their interplay significantly influences clinical outcomes, consistent with existing literature on the subject.​​​​​​​

Limitations 

Several limitations of our study include reliance on single-center data, a retrospective design prone to recall bias, the absence of longitudinal data, and potential unaccounted confounders, limiting generalizability and causal inference. 

## Conclusions

Our study emphasizes the multifaceted nature of cardiovascular disease and its associations with chronic kidney disease. It underscores the significance of risk factor management, early detection, and comprehensive assessment of both cardiac and renal health in improving patient outcomes. Further research is warranted to explore potential interventions aimed at mitigating the synergistic impact of CVD and CKD on patient morbidity and mortality. The significant implications of our study highlight the need for a comprehensive approach to managing patients with both cardiovascular disease and chronic kidney disease. The identified predictors of renal dysfunction and CAD severity can assist in risk assessment and personalized treatment strategies, ultimately improving patient outcomes.

## References

[1] Tsao CW, Aday AW, Almarzooq ZI (2022). Heart disease and stroke statistics-2022 update: A report from the American Heart Association. Circulation.

[2] Sarnak MJ, Levey AS, Schoolwerth AC (2003). Kidney disease as a risk factor for development of cardiovascular disease: a statement from the American Heart Association Councils on Kidney in Cardiovascular Disease, High Blood Pressure Research, Clinical Cardiology, and Epidemiology and Prevention. Circulation.

[3] Hindawi Hindawi (2022). Prevalence of Cardiovascular Disease and Associated Risk Factors among Adult Population in the Gulf Region: A Systematic Review. https://www.hindawi.com/journals/aph/2015/235101/.

[4] Fox CS, Larson MG, Leip EP, Culleton B, Wilson PW, Levy D (2004). Predictors of new-onset kidney disease in a community-based population. JAMA.

[5] Klag MJ (19972022). End-stage renal disease in African-American and white men: 16-Year MRFIT findings. JAMA [Internet.

[6] Glassock RJ, Rule AD (2012). The implications of anatomical and functional changes of the aging kidney: with an emphasis on the glomeruli. Kidney Int.

[7] Parikh NI, Hwang SJ, Larson MG, Meigs JB, Levy D, Fox CS (2006). Cardiovascular disease risk factors in chronic kidney disease: overall burden and rates of treatment and control. Arch Intern Med.

[8] Mousa D, Alharbi A, Helal I (2021). Prevalence and associated factors of chronic kidney disease among relatives of hemodialysis patients in Saudi Arabia. Kidney Int Rep.

[9] Baigent C, Burbury K, Wheeler D (20002022). Premature cardiovascular disease in chronic renal failure. Lancet [Internet.

[10] Collins AJ, Kasiske B, Herzog C (2005). Excerpts from the United States Renal Data System 2004 annual data report: atlas of end-stage renal disease in the United States. Am J Kidney Dis.

[11] Liu JH, Lin SY, Hsu CY, Lin HH, Liang CC, Sung FC, Huang CC (2012). The risk for chronic kidney disease in patients with heart diseases: a 7-year follow-up in a cohort study in Taiwan. BMC Nephrol.

[12] Milane A, Khazen G, Zeineddine N (2015). Association of coronary artery disease and chronic kidney disease in Lebanese population. Int J Clin Exp Med.

[13] Gao Z, Chen Z, Sun A, Deng X (2019). Gender differences in cardiovascular disease. Medicine in Novel Technology and Devices.

[14] Carrero JJ, Hecking M, Chesnaye NC, Jager KJ (2018). Sex and gender disparities in the epidemiology and outcomes of chronic kidney disease. Nat Rev Nephrol.

[15] McCullough PA, Li S, Jurkovitz CT (2008). Chronic kidney disease, prevalence of premature cardiovascular disease, and relationship to short-term mortality. Am Heart J.

[16] Mallappallil M, Friedman EA, Delano BG, McFarlane SI, Salifu MO (2014). Chronic kidney disease in the elderly: evaluation and management. Clin Pract (Lond).

[17] Yang Y, Peng N, Chen G (2022). Interaction between smoking and diabetes in relation to subsequent risk of cardiovascular events. Cardiovasc Diabetol.

[18] Kitsios GD, Dahabreh IJ, Trikalinos TA, Schmid CH, Huggins GS, Kent DM (2011). Heterogeneity of the phenotypic definition of coronary artery disease and its impact on genetic association studies. Circ Cardiovasc Genet.

[19] Albert NM, Tyson RJ, Hill CL (2021). Variation in use and dosing escalation of renin angiotensin system, mineralocorticoid receptor antagonist, angiotensin receptor neprilysin inhibitor and beta-blocker therapies in heart failure and reduced ejection fraction: Association of comorbidities. Am Heart J.

[20] Hubers SA, Brown NJ (2016). Combined angiotensin receptor antagonism and neprilysin inhibition. Circulation.

[21] Jankowski J, Floege J, Fliser D, Böhm M, Marx N (2021). Cardiovascular disease in chronic kidney disease: Pathophysiological insights and therapeutic options. Circulation.

[22] Lee S, Kang S, Joo YS (2021). Smoking, smoking cessation, and progression of chronic kidney disease: results from KNOW-CKD study. Nicotine Tob Res.

[23] Beldhuis IE, Lam CS, Testani JM, Voors AA, Van Spall HG, Ter Maaten JM, Damman K (2022). Evidence-based medical therapy in patients with heart failure with reduced ejection fraction and chronic kidney disease. Circulation.

[24] Ducrocq G, Amarenco P, Labreuche J (2013). A history of stroke/transient ischemic attack indicates high risks of cardiovascular event and hemorrhagic stroke in patients with coronary artery disease. Circulation.

